# Cerebral Folate Metabolism in Post-Mortem Alzheimer’s Disease Tissues: A Small Cohort Study

**DOI:** 10.3390/ijms24010660

**Published:** 2022-12-30

**Authors:** Naila Naz, Syeda F. Naqvi, Nadine Hohn, Kiara Whelan, Phoebe Littler, Federico Roncaroli, Andrew C. Robinson, Jaleel A. Miyan

**Affiliations:** 1 Division of Neuroscience, School of Biological Sciences, Faculty of Biology, Medicine & Health, The University of Manchester, 3.540 Stopford Building, Oxford Road, Manchester M13 9PT, UK; 2 Geoffrey Jefferson Brain Research Centre, Manchester Academic Health Science Centre (MAHSC), The University of Manchester, Salford Royal Hospital, Salford M6 8HD, UK

**Keywords:** Alzheimer’s disease, cerebral folate, folate metabolism, cerebrospinal fluid, ALDH1L1, astrocytes, neurons, methylation

## Abstract

We investigated the cerebral folate system in post-mortem brains and matched cerebrospinal fluid (CSF) samples from subjects with definite Alzheimer’s disease (AD) (*n =* 21) and neuropathologically normal brains (*n =* 21) using immunohistochemistry, Western blot and dot blot. In AD the CSF showed a significant decrease in 10-formyl tetrahydrofolate dehydrogenase (FDH), a critical folate binding protein and enzyme in the CSF, as well as in the main folate transporter, folate receptor alpha (FRα) and folate. In tissue, we found a switch in the pathway of folate supply to the cerebral cortex in AD compared to neurologically normal brains. FRα switched from entry through FDH-positive astrocytes in normal, to entry through glial fibrillary acidic protein (GFAP)-positive astrocytes in the AD cortex. Moreover, this switch correlated with an apparent change in metabolic direction to hypermethylation of neurons in AD. Our data suggest that the reduction in FDH in CSF prohibits FRα-folate entry via FDH-positive astrocytes and promotes entry through the GFAP pathway directly to neurons for hypermethylation. This data may explain some of the cognitive decline not attributable to the loss of neurons alone and presents a target for potential treatment.

## 1. Introduction

Alzheimer’s disease (AD) is an irreversible neurodegenerative condition affecting around 40 million people over the age of 60, with numbers reportedly doubling every 20 years, worldwide [1]. More than 2000 clinical trials, aimed to slow or halt the disease have failed [2,3,4,5]. These trials were based on different hypotheses of disease aetiology, pathogenesis and progression and likely failed because intervention happened too late in the course of the disease. Given the personal, social and economic burden of AD, there is an urgent need to identify novel approaches to the condition. Cerebral folate metabolism is one potential new and promising target.

Folate is absorbed across the gut and transported to the whole body by folate receptor alpha (FOLR1 or FRα) which is the main transporter and carries folate across the choroid plexus into the cerebrospinal fluid (CSF) [6]. Aldehyde dehydrogenase-1L1 (ALDH1L1), also known as 10-formyl tetrahydrofolate dehydrogenase (FDH), is a key folate binding protein and enzyme involved in many functions including tumour suppression [7,8,9,10]. Specific forms of epilepsy, a form of autism and a related severe neurological condition, named cerebral folate deficiency syndrome (CFD) are caused by maternal autoantibodies to FRα that block the transfer of folate from foetal blood into the foetal brain leading to profound folate deficiency and resulting in poor development and progressively severe neurological signs and symptoms after birth [11,12,13,14,15,16,17,18]. Furthermore, CSF drainage impairment has been directly linked to cerebral folate deficiency or imbalance with the extreme case of hydrocephalus showing a blockade of available folate in the CSF by the withdrawal of the folate-binding protein, FDH from CSF [19].

Metabolic deficiencies have been highlighted as one of the potential causes of AD [2]. Our recent study demonstrated that single nucleotide polymorphisms (SNPs) affecting the folate enzyme methylene tetrahydrofolate dehydrogenase 1 (MTHFDH1) were significantly associated with AD and were also found to have resulted in a shift in folate metabolism [20]. Folate deficiency either due to CFD or folate imbalance resulting from CSF drainage insufficiency is associated with a multitude of conditions including AD. Silverberg and colleagues demonstrated both reduced CSF production as well as enlargement of the ventricles in Alzheimer’s patients [21,22,23]. In addition, they found that CSF turnover rate decreases in normal ageing associated with amyloid deposition [24]. In other neurological conditions, including benign external hydrocephalus associated with autism, the large head size is associated with CSF accumulation both outside the brain, in the subarachnoid space [25,26,27], and also inside the brain associated with enlarged ventricles [25,28]. Significantly, disease severity has also been associated with increased ventricular enlargement in AD [29,30,31,32] indicating a potential common mechanism in conditions affecting the cerebral cortex and suggesting a common CSF turnover and cerebral folate metabolic link to many cerebral conditions. This is reinforced by a recent report detailing changes in methylation as well as changes in polyamine pathways in AD, that are intimately linked to folate metabolism [33], as well as the critical review of Liu et al. (2019) [2] and our own studies identifying an abnormality in a gene for a folate enzyme [20]. The current study, therefore, investigated the cerebral folate system in post-mortem brains and matched CSF of those considered neuropathologically normal for age (Braak stage 0-II) and those with neuropathologically confirmed AD (Braak stage V-VI). We, and others, have previously shown that FRα and FDH are involved in the transport and exchange of folate across the blood-CSF barrier and delivery to the brain across the CSF-brain barrier [34,35,36,37]. We have also shown that reduced FDH in CSF is associated with a cell cycle arrest in neural stem/progenitor cells in hydrocephalus [38,39]. We therefore initially focused on these molecules and aimed to identify any changes in the supply, transport and metabolism of folate in the AD group as compared to neuropathologically normal control group.

## 2. Results

### 2.1. Cerebrospinal Fluid (CSF) Analysis

**Figure 1:**
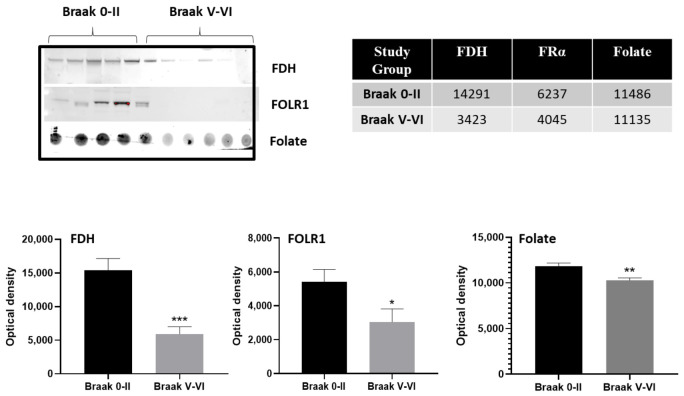
Western and dot blot analysis of CSF for FDH, FRα and folate. The images of the relevant bands and dots from different blots for FDH, FRα and folate are shown at top with optical density measurements plotted as mean ±SEM for *n* = 21 per group. *, ** and *** show significance at *p* < 0.05, 0.001, and 0.0001, respectively. The mean optical density measurements are shown in the table. Images of the full blot membranes are shown in Supplementary Figure S1.

Western blot analysis of CSF from the AD group (Braak V-VI) demonstrated a significant decrease in FDH protein expression as compared to the neurologically normal group (Braak 0-II; Figure 1).

FDH was significantly reduced in AD compared to normal with a statistical significance of *p* ≤ 0.0001. Similarly, FRα (*p* = 0.0308) and folate (*p* = 0.0011) were also significantly reduced in AD (Figure 1). The raw optical density values for each parameter studied are given in tabular form in Figure 1. This reduction in folate related proteins and metabolites indicates a decreased folate supply, by FRα transport from the blood across the choroid plexus, as well as a general downregulation in folate metabolism in CSF of AD patients. Full gels and membranes are illustrated in Supplementary Figure S1.

### 2.2. Immunohistochemistry for Cerebral Cortex

**Figure 2:**
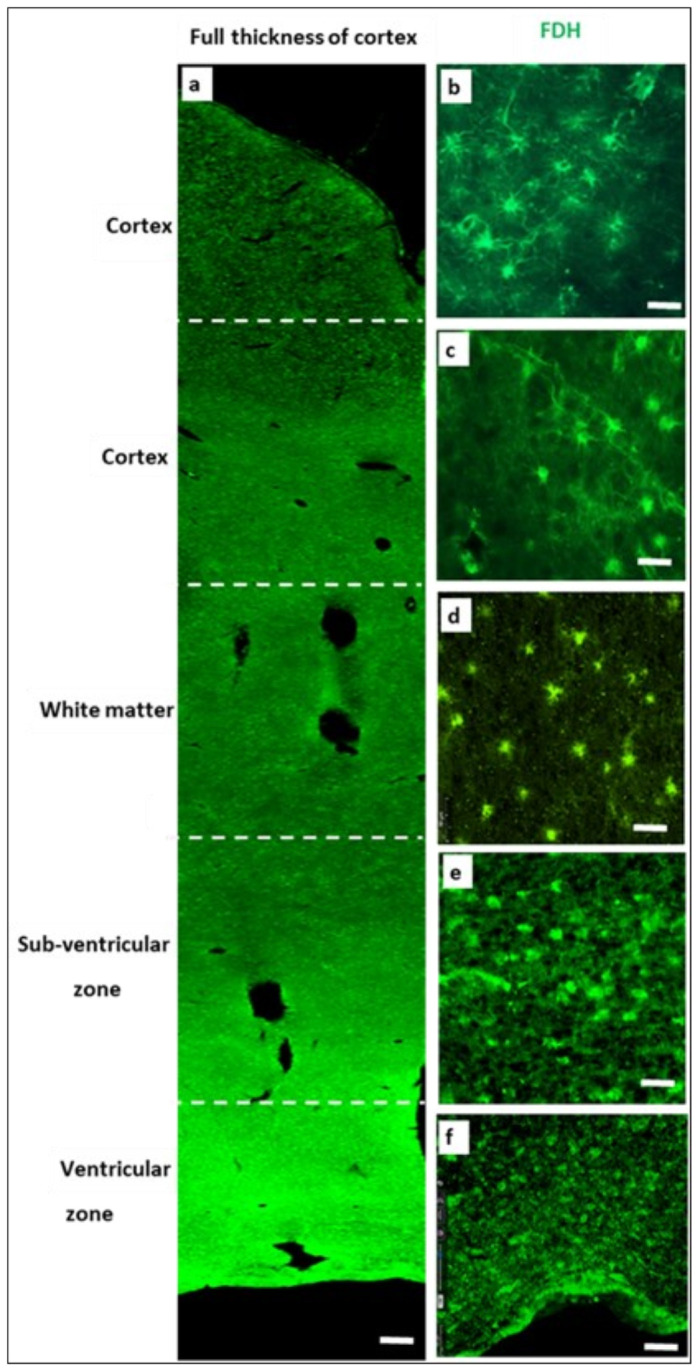
Immunofluorescence staining of Braak 0-II brain sections for FDH (green). Left panel (**a**) shows the entire section of cortex shown at low power (5×; scale bar: 2 mm) to orientate the different regions of brain shown in the right panels (**b**–**f**) at 200×, scale bar: 50 µm. (**b**) Cortex near the pia mater, (**c**) Mid cortex region, (**d**) White matter region, (**e**) sub-ventricle zone, (**f**) ventricular zone. The image is representative of *n =* 3 brains.

In normal brain, FDH-positive cells form a network from the top of the cortex, where they connect to the pia mater (Figure 2a) right through to the ependymal lining of the ventricles. These FDH^+^ cells have different morphologies in different cortical regions; classical stellate morphology in the cortex (Figure 2b,c), small and thin with short processes in the white matter (Figure 2d), large and more rounded with short processes in the sub-ventricular zone (Figure 2e) and with longer processes in the ventricular zone (Figure 2f) looking more like the classical stellate morphology.

**Figure 3:**
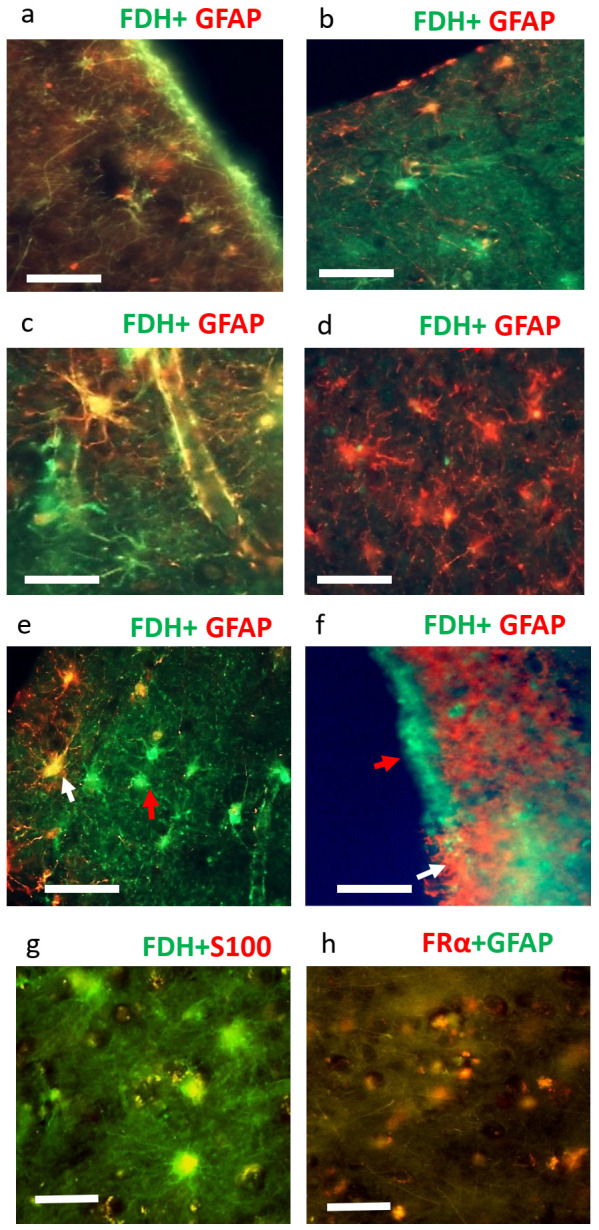
Immunofluorescence staining of Braak 0-II brain for FDH, GFAP, FRα and GFAP. Double immunofluorescence for FDH (green) and GFAP (red) staining of neurologically normal brain in cortical region near the pia mater (**a**,**b**) 100×, scale: 100 µm, white matter region (**c**), sub ventricular zone (**d**) and ventricular zone (**e**,**f**) at 200×, scale: 50 µm. The white arrows indicate FDH^+^/GFAP^+^ whereas red arrow indicates FDH^+^/GFAP^−^. Double immunofluorescence staining of FDH (green) and S100 (red) (**g**) to demonstrate FDH^+^ /S100^+^ astrocytes 200×, scale: 50 µm. Double immunofluorescence staining for FRα and GFAP (**h**, 100×; scale bar 100 µm). The figure is representative of *n =* 3.

Since FDH is a recognised marker of astrocytes, the nature of these FDH^+^ cells was further confirmed by co-localisation with the astrocyte markers S100 and GFAP (Figure 3). Both markers have variable intensities of co-localisation. Moreover, they share selective sub-cellular co-localisation with FDH^+^ astrocytes indicating changes between markers potentially depending on localised functional requirements. To elaborate, adjacent areas of cortex, associated with the pia mater, were observed to be FDH^+^/GFAP^−^ (Figure 3a) or FDH^−^/GFAP^+^ (Figure 3b). In other parts of the cortex, GFAP^+^/FDH^+^ astrocytes were observed with their GFAP^+^/FDH^+^ end feet over the surface of a capillary (Figure 3c). Next to it, FDH^+^/GFAP^−^ were observed with FDH^+^/GFAP^−^ end feet on the surface of a neighbouring capillary (Figure 3c). Additionally, areas of cortex (Figure 3b) and ependymal (Figure 3e) demonstrated very few GFAP^+^/FDH^+^ astrocytes. Similar to these observations in the cortical marginal zone, we found regions of the ventricular zone and ependyma that had adjacent areas of FDH^+^/GFAP^−^ or FDH^−^/GFAP^+^ glial processes (Figure 3f). In the cortex, the FDH^+^ cells also demonstrated colocalisation with S100 (Figure 3g). FRα and GFAP demonstrated limited colocalisation most notably in the marginal zone of the cortex (Figure 3h). Micrographs taken with each separate wavelength for this, and subsequent figures, are shown in Supplementary Figures S2–S6.

**Figure 4:**
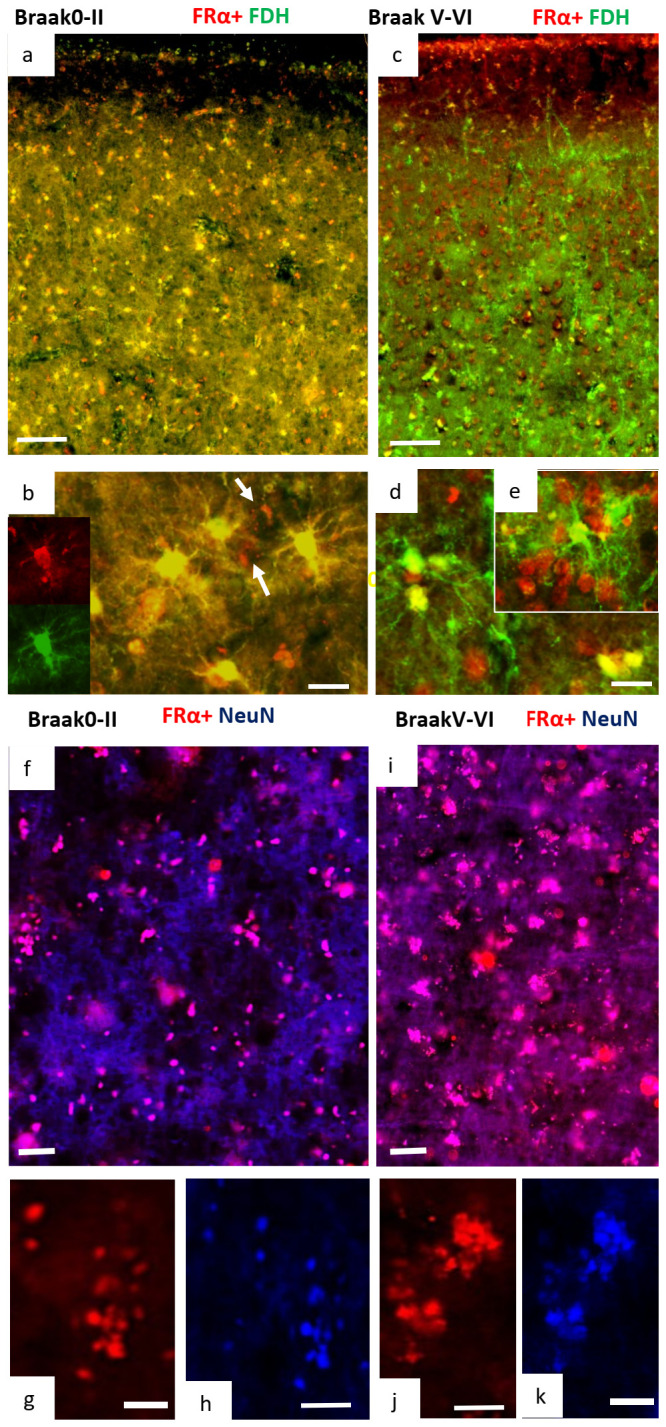
Immunofluorescence staining for FDH, FRα and NeuN. Double immunofluorescence staining for FRα (red) and FDH (green) in Braak 0-II (**a**,**b**) and Braak V-VI (**c**–**e**) brains ((**a**,**c**) 100×, scale:100 µm and (**b**) 400×, 20 µm). Co-localisation (yellow) of FRα with FDH in the FDH-positive astrocytes in normal brains (**a**,**b**) shows almost complete separation in Alzheimer brain (**c**–**e**). FRα appears as speckled red staining in normal neuronal cell bodies (white arrows in (**b**)) while in Alzheimer cortex these cells are full of red, FRα (**d**,**e**). Double immunofluorescence staining for FRα (red) and NeuN (blue) in normal (**f**) and AD (**i**) brains show colocalisation, which is shown in separate channels in (**g**,**h**) and (**j**,**k**) ((**f**), 200×, scale: 50 µm ((**g**,**h**,**j**,**k**), 400×, scale: 20 µm). The figure is representative of neurologically normal, Braak 0-II *n* = 3 and AD, Braak V-VI *n* = 4.

Double immunostaining for FDH and FRα in normal and AD brains showed a striking and substantial change in distribution of these two folate related proteins (Figure 4). In normal brains, cortical cells were found to be FDH^+^/FRα^+^ with a stronger colocalisation in cells extending to the pia mater (Figure 4a). Additionally, FRα staining appeared as speckles within what we believe to be neuronal cells (white arrows in Figure 4b) reflecting its transport through endocytic vesicles where the FDH^+^/FRα^+^ astrocytes also appeared to have close associations with these (Figure 4b). In contrast, in AD brain, most cells were either FDH^+^/FRα^−^ or FDH^−^/FRα^+^ indicating that the cortical expression of FRα and FDH has become almost completely separated. Very few cells maintain FDH^+^/FRα^+^ indicating that only a few FDH^+^ astrocytes still have positive staining for FRα (Figure 4c–e) and thus retain transport capability for FRα and folate. The FDH^+^ astrocytes in AD brain have more extensive and denser processes than in normal brain (Figure 4b,d). To identify the other cell type positive for FRα, double immunofluorescence staining with the neuronal nucleus marker (NeuN) was performed. In normal brain cortex, a few cells were found to be FRα^+^/NeuN^+^, indicating FRα related folate supply is probably low to neuronal cells (Figure 4f–h). However, in AD brain, many cortical cells were seen to be FRα^+^/NeuN^+^ (Figure 4i,k) indicating that FRα is concentrated into neuronal cell bodies throughout the cortex in AD.

**Figure 5:**
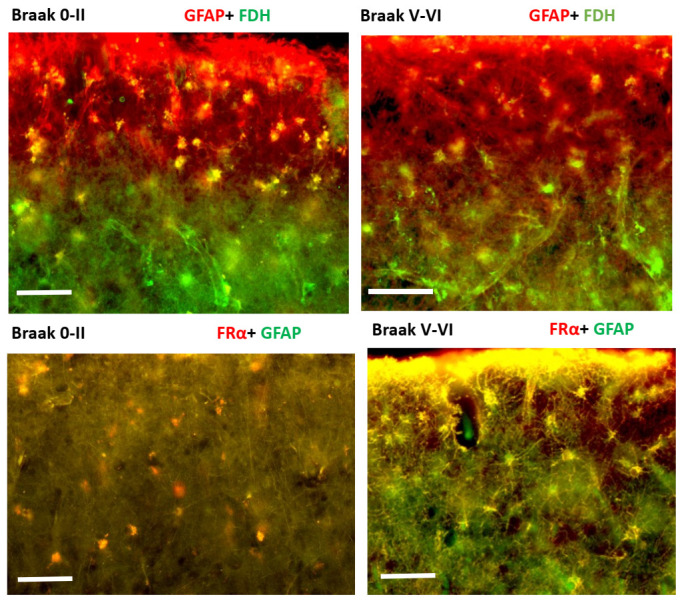
Immunofluorescence staining for GFAP, FRα and FDH. Upper panel: Double immunofluorescence staining for FDH (green) and GFAP (red) in normal and AD brains. Co-localisation (yellow) is observed in astrocytes. Lower panel: Double immunofluorescence staining for FRα (red) and GFAP (green) in neurologically normal and AD brains. Co-localisation is observed as bright yellow colour. Magnification 200×, scale bar: 50 µm. The figure is representative of neurologically normal *n =* 3 and AD brains *n =* 4.

In normal brain, a strong expression of GFAP^+^/FDH^+^ in cells was observed with a few cells FRα+/GFAP+ indicating that most GFAP positive astrocytes were strongly positive for FDH and least positive for FRα. In contrast, in AD brain, although some cells were FDH^+^/GFAP^+^ but mostly cells were found to be FRα^+^/GFAP^+^ indicating a clear switch in signal from FDH positive astrocytes to FRα positive astrocytes. This switch was more obvious near to the pia mater in cortex (Figure 5).

**Figure 6:**
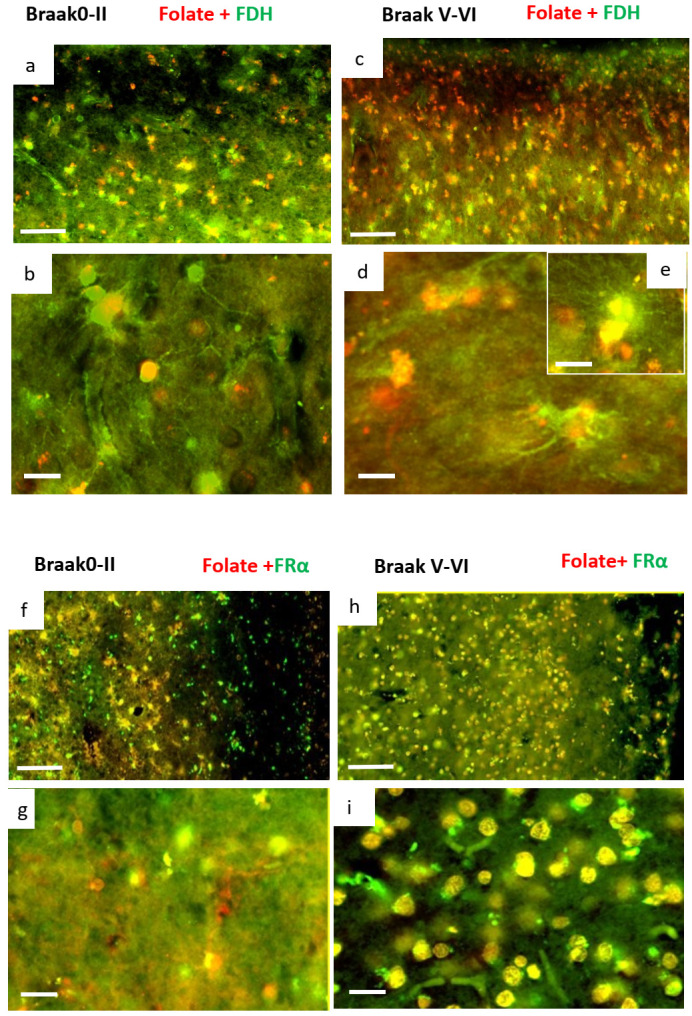
Immunofluorescence staining for folate, FRα and FDH. Double immunofluorescence staining for FDH (green) and folate (red) in neurologically normal ((**a**) 100 × 100 µm and (**b**) 400 × 20 µm) and AD brain ((**c**) 100 × 100 µm and (**d**) 400 × 20 µm). Some co-localisation (yellow) of folate and FDH ((**e**) 400 × 20 µm) is observed. Double immunofluorescence staining for FRα (green) and folate (red) in normal ((**f**) 100 × 100 µm and (**g**) 400 × 20 µm) and AD brain ((**h**) 100 × 100 µm and (**i**) 400 × 20 µm). The figure is representative of neurologically normal *n =* 3 and AD *n =* 4 brains.

In the normal brain, most of the cortical cells are FDH^+^/folate^+^ (Figure 6a,b) and FRα^+^/folate^−^ (Figure 6f,g) indicating folate transport by FRα through the FDH^+^ network of astrocytes. By contrast, in AD brain, cortical cells appeared to have two set of populations FDH^−^/Folate^+^ and a few FDH^+^/Folate^+^ (Figure 6c,d)^.^ A large number of cells were found to be FRα^+^/folate^+^ (Figure 6h,i) which seems to be correlated with the FDH^−^/Folate^+^ cells (Figure 6c–e). Overall, this data set indicated a switch in folate transport pathway from FDH^+^/FRα^+^/folate^+^/GFAP^−^ astrocytes in normal brain to FRα^+^/folate^+^/GFAP+ astrocytes that are associated with NeuN^+^ neurons in AD brain.

### 2.3. Changes in Methylation in Neuronal Cells of Alzheimer’s Disease Brain

**Figure 7:**
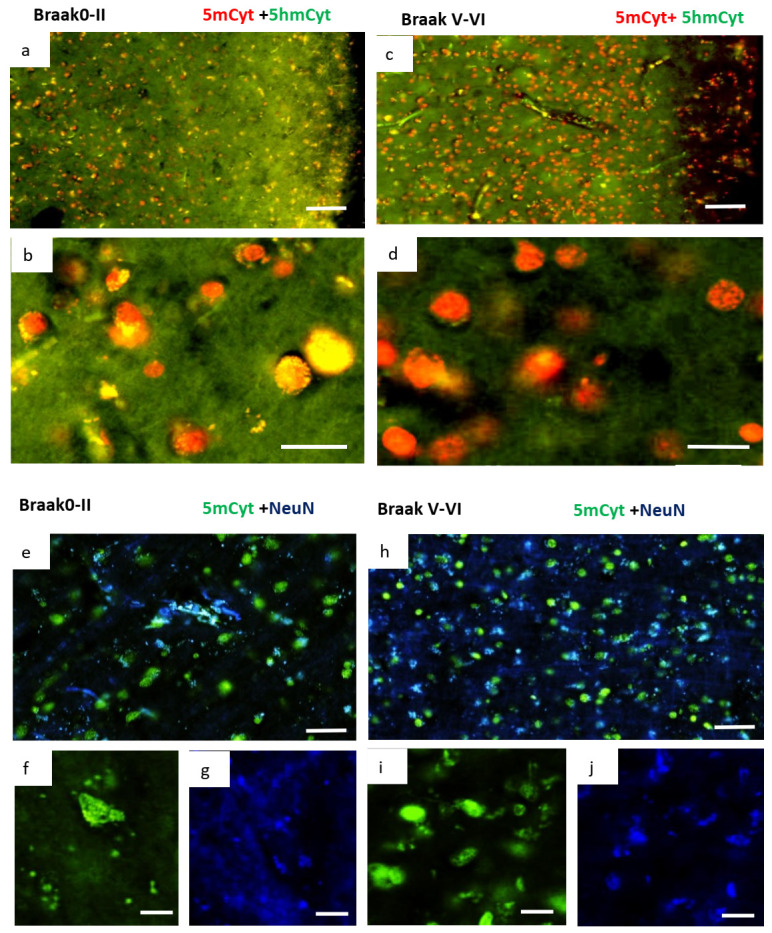
Immunofluorescence staining for 5-methyl cytosine (5mc, marker of methylation) and 5-hydroxy methyl cytosine (5hmc, marker of demethylation) in neurologically normal (Braak 0-II) and AD (Braak V-VI) brains. Double immunofluorescence staining for 5-methyl cytosine (red) and 5-hydroxy methyl cytosine (green) in normal cortical cells ((**a**) at 100×, scale: 100 µm and (**b**) 400×, scale: 20 µm) show a balance of methylation and demethylation (seen as colocalised, yellow staining). In AD brain ((**c**) at 100×, scale: 100 µm and (**d**) 400×, scale: 20 µm) cells show almost exclusive methylation with very little demethylation. Double immunofluorescence staining for 5mc (green) and the neuronal marker NeuN (blue) in normal (**e**) and AD (**h**) are also shown in separated channels (**f**,**g**,**i**,**j**) to show that many cells have colocalised with many more in AD brain, indicating hypermethylation of neurones in AD. (**e**,**h**), at 100×, scale: 100 µm. (**f**–**j**) at 200×, scale: 50 µm. The figure is representative of neurologically normal *n =* 3 and AD *n =* 4 brains.

In the normal brain, with some regional differences, most of the cortical cell population was positive for both 5mC and 5hmC. (Figure 7a,b). In contrast, AD brain cortical cells demonstrated very little colocalisation of 5mC and 5hmC. Essentially, most cells were found to be 5mC^+^/5hmC^−^ (Figure 7c,d). This was particularly evident in cells near the pia mater. This pattern of 5mC^+^/5hmC^−^ was in line with FDH^−^/FRα^+^/folate^+^/NeuN^+^ cells near the pia mater as shown in Figure 4. The 5mC^+^ cells demonstrated colocalisation with the neuronal marker NeuN in normal and AD (Figure 7e–j). The data indicate that in AD brain, the cortical cells, particularly neurons, are hypermethylated, presumably due to FRα related transport of folate into the nuclei of neuronal cells.

## 3. Discussion

In the present study, western and dot blot analysis of CSF demonstrate potentially important changes in cerebral folate supply and transport, where a significant decrease in FDH is evident together with a tendency of raised FRα and folate, perhaps reflecting reduced uptake and use, is seen in AD CSF. These results observed in AD are similar to our previous observations in hydrocephalus [19,36,37]. The reduction in FDH is becoming established as a hallmark of CSF drainage insufficiency and may therefore suggest a similar association with CSF drainage capacity, ventricular dilatation and disease severity in AD [29,30,40]. With ventricular enlargement and/or CSF accumulation found as hallmark features of many conditions including dementia and AD, Autism and Schizophrenia, depression and bipolar [28,29,32,41,42,43,44,45,46,47,48,49,50,51,52,53,54,55,56,57,58,59,60,61,62,63], a cerebral folate issue may also be present as we have found in early stages of hydrocephalus [19]. Indeed, some of these conditions respond to folate treatments [64,65,66,67,68,69,70,71,72]. Even though Silverberg and colleagues suggest a decrease in CSF output in ageing, they also describe raised CSF pressure and accumulation of fluid in AD [39,73] indicating that CSF drainage may be the most significant factor as is also suggested by the reduced FDH found in this study. AD is not associated with raised intracranial pressure or hydrocephalus but an association between disease severity and ventricular enlargement has been reported and may be an early marker of the development/progression of this condition [50,74,75]. Reduced FDH would eventually result in reduced availability of folate to the brain. We believe these changes in folate related proteins in CSF are physiological changes rather than dilution effects of increased/increasing fluid since other proteins and metabolites remain at normal or above normal levels, e.g., for FRα and folate (other data in preparation for publication).

In the current study, we established how FDH^+^ astrocytes are distributed throughout the adult brain, forming a network stretching from the pia mater of the cerebral cortex to the ventricular zone. Although FDH has been identified as an astrocyte marker, the current study is the first to describe this unique network of cells we believe is involved in folate delivery. Folate supply in the developing brain is from the CSF into FDH^+^ radial glia [19]. As gliogenesis progresses in later development, then several types and forms of astroglia appear throughout the cortex [76] and the radial glia lose their connection to the pia mater to become ependymal cells. As the cortex enlarges, the FDH cells provide essential folate for DNA synthesis, methylation, neurotransmitter and nitric oxide synthesis, myelination as well as other essential metabolic pathways [20]. The normal feeder for this network is FDH in the CSF that binds to folate and FRα and this is required for transport across the ependymal barrier from the ventricles, and the pia mater from the subarachnoid space [19,77]. Where CSF dynamics and drainage are abnormal, FDH is not secreted into the CSF and the brain is deprived of folate through this route as we have found in hydrocephalus. The situation in AD indicates that a similar folate block exists through decreased FDH in the CSF. However, FRα seems able to transport folate into the adult affected brain through a different, perhaps less specific route, the GFAP astrocyte network. In this case, metabolism is also different with hypermethylation occurring in the affected brain regions examined in this study.

A significant finding in normal brain is that the pia mater and marginal zone seem to be essential for folate transfer from CSF into the cortex giving the subarachnoid CSF a vital function in delivering this critical metabolite [77]. In the marginal zone, FDH-positive astrocytes are significantly associated with the main blood vessels entering the brain. These vessels are surrounded by Virchow-Robin spaces which are filled with CSF so that the astrocytes are in contact with the CSF in these compartments most recently associated with the glymphatic pathway [78,79,80]. Interestingly, these vessels are also the site for glymphatic fluid transfer into the brain parenchyma so that the FDH^+^ astrocytes may be involved in this process as well as other astrocyte functions. FDH^+^ as well as other astrocytes are also associated with the capillary network throughout the brain.

Previous studies have reported that folate is transported across the choroid plexus into the CSF by FRα with little, if any, transport across the endothelial blood–brain barrier [81]. Although positive staining for reduced folate carrier (RFC) in cortical endothelium and neurons has been reported (Human Protein Atlas: https://www.proteinatlas.org/, accessed on 7 September 2021) and RFC is elevated in mice missing FRα [81] but this does not seem to happen where FRα is blocked later in life [12,16,66,82]. In our present study, we demonstrated that within normal brain, the FDH^+^/FRα^+^/GFAP^−^/folate^+^ cells seem to form the main pathway for folate delivery. It seems likely that both FRα and FDH, perhaps bound together by folate, carry folate into the brain through this pathway. Thus, FDH must be synthesised by the cells, secreted into the CSF and then reabsorbed when bound to folate or folate+ FRα.

In the AD brain, an increase in FRα^+^/GFAP^+^/folate^+^ cells indicates that a different pathway opens to FRα-folate through GFAP-positive astrocytes. An increased expression of FRα^+^/GFAP^+^/folate^+^ cells in the brain of AD is also in line with a decrease in CSF levels of FRα and folate, though the decrease we found was not significant [81]. Similar to our findings in hydrocephalus, there does seem to be more FDH in the AD brain tissue seen in the density of FDH^+^/GFAP^+^ astrocyte processes as well as a greater intensity of staining. This may be a consequence of greater synthesis and expression of FDH by the astrocytes and/or by the inhibition of secretion into the CSF [19,66,72,82,83,84,85,86,87]. Consequently, a decrease in FDH in AD CSF would have the effect of preventing FRα uptake into the FDH astrocyte network, leading to the changes we observe in the AD brain. It seems possible that the loss of FDH in the CSF, and associated changes observed in the Alzheimer’s brain, may also contribute to glymphatic impairment and the build-up of toxins in the brain including tau and amyloid [80,88,89,90,91,92,93,94].

In AD brain, an increase in 5mc indicates enhanced methylation in NeuN^+^ neurons and is in line with increased FRα^+^/folate^+^/NeuN+ cells that were also associated with the FDH^+^/folate^+^ astrocytes processes indicating enhanced accumulation of FRα bound folate into the neurons. FDH-bound folate transported in the astrocytes is also feeding the neurons and, as a consequence, neuronal cells are in a state of hypermethylation. The literature on AD brains is contradictory with some finding hypomethylation and some hypermethylation. These differences may reflect the different areas/subareas being analysed and one might speculate that those areas essential for function may be hypomethylated, while those not essential are being shut down by hypermethylation to protect against the neurodegenerative processes in AD. The folate changes we have found need more detailed analysis to establish their roles in cause, aetiology and progression of the disease.

## 4. Materials and Methods

### 4.1. Brain Tissue

All brain tissues were supplied from the Manchester Brain Bank under their ethical approval (09/H0906/52+5 and 19/NE/0242) for collection and use of human tissue in neurodegenerative disease research. In this study, we were provided with formalin-fixed tissue from the temporal cortex that included the full thickness of the cortex from the pia mater to the ventricular ependymal. Post-mortem CSF samples were also provided from the same brains. Case information is given in Table 1a,b.

### 4.2. Western and Dot Blots

For relative measures of CSF proteins, western blot analysis of CSF was performed comparing normal (*n =* 21) and AD (*n =* 21). Samples were prepared by mixing 6 µL of CSF with Laemmli sample buffer (Bio-Rad Labs Ltd., Watford, UK) and 2-mercaptoethanol (Sigma-Aldrich, Gillingham, UK) then heated to 70 °C for 10 min before cooling and loading into Nupage precast SDS polyacrylamide gels (Life Technologies, Paisley, UK). 150 v were applied to the gels to separate the proteins over a 40–60 min run. Proteins were transferred onto nitrocellulose membranes using an iBlot semi-dry transfer system (Life Technologies, Paisley, UK). Blots were then placed in a blocking buffer (5% BSA in PBS with 1% tween 20) for an hour before being probed with a primary antibody to the specific protein of interest (Table 2) in the same blocking buffer, at a common dilution of 1:3000, overnight at 4 °C. Blots were washed in PBS with 0.1% tween 20 and then incubated in secondary antibody conjugated to HRP for 2 h at room temperature before a further wash. Membranes were then incubated in ECL solution (Bio-Rad Labs Ltd., Watford, UK) for 5 min before exposure on a LiCor C-digit scanner (Li-Cor Biotechnology Ltd., Cambridge, UK). Scans were collected into LiCor Image Studio software for quantification and analysis. For dot blots, 6 µL of CSF from normal (*n =* 21) and AD (*n =* 21), were placed directed on nitrocellulose membranes and allowed to spread and dry at room temperature for an hour. They were then blocked, and the remaining procedure was as described for Western blots. All antibodies used in this study are listed in Table 2.

### 4.3. Immunohistochemistry

The formalin-fixed superior and medial temporal gyrus from subjects with definite AD (*n =* 4) and normal subjects showing mild ageing-related changes (*n =* 3) were obtained from the Manchester Brain Bank. The samples included the arachnoid, cortex, white matter and ventricular wall. The tissue specimens were washed and incubated in 30% sucrose solution (for cryoprotection) for several days until they sank. They were then frozen in isopentane cooled with dry ice before mounting on a stub and locating into the chuck of a Leica DM1900 cryostat (Leica Microsystems Ltd., Milton Keynes, UK). 50 µm thick sections were obtained which spanned from pia mater to the ependyma. Sections were collected as free-floating rather than onto glass slides to ensure optimal immuno staining. Heat-induced epitope retrieval was performed in pre-heated sodium citrate buffer (pH 6.0) microwaved for 5–10 s and kept in the cooling solution for half an hour. Sections were then incubated in blocking buffer, consisting of 1% Triton-X100 (Sigma-Aldrich, Gillingham, UK) in Phosphate-buffered saline (PBS) containing 0.5% goat serum and 0.5% donkey serum, and then incubated in primary antibodies (listed in Table 2) diluted in the same blocking buffer overnight at 4 °C. Washing in PBS with 1% Tween-20 (Sigma-Aldrich, Gillingham, UK) was followed by incubation in Alexa Fluor conjugated secondary antibodies (Table 2) in the same solution. After further buffer washes to remove unbound antibodies, the sections were placed onto glass slides and mounted in Fluoroshield (Abcam, Cambridge, UK) containing 4′,6-diamidino-2-phenylindole (DAPI) to stain nuclei. Coverslips were sealed around the edges with clear nail varnish to prevent movement during scanning. Sections were scanned on a 3D Histech Pannoramic 250 Flash Slide Scanner (3DHistech Ltd., Budapest, Hungary) before viewing on 3D Histech Caseviewer software.

### 4.4. Statistical Analysis

Adjusted band volume and dot volume intensities were obtained using ImageLab software by subtracting the background intensity for each blot image from the band or dot volume of each individual. Adjusted band volume was averaged across repeat experiments and presented in arbitrary units of optical density (OD). GraphPad Prism Version 9.2.0 was used to analyse the differences between average adjusted band volumes of normal compared to AD. Normality tests were not required due to the small number of individuals in each group, thus parametric tests were robust enough in this circumstance. All groups were tested for equal variance with an F test. Data sets with equal variance were analysed using a Student’s unpaired *t*-test. Those that failed to show equal variance had a Welch correction applied to this t-test. All data were presented as mean ± standard error of the mean (±SEM) and to 4 decimal places. Statistical significance was expressed at the *p* < 0.05 level throughout.

## 5. Conclusions

The current study has identified a potentially important change in folate supply to the AD brain. We postulate that with a decrease in the folate binding protein, FDH in the CSF, there is a switch in folate supply from the FRα-FDH pathway to the FRα-GFAP pathway. The consequence of this switch seems to be a change in metabolism to hypermethylation where FRα, with folate, ends up in the neurons of the cortex and the folate is delivered to the nuclei where methylation occurs. We further suggest that this may be a strategy to shut down all but essential activity to safeguard surviving neurons from the toxic effects of AD. This may in turn explain some of the cognitive decline not attributable to the loss of neurons alone. These findings present strong evidence for a larger and more detailed study of cerebral folate metabolism in AD.

### Limitations of the Current Study

The biggest limitation of this study was in the use of post-mortem tissues and matched post-mortem CSF samples. In addition, we only included 21 individuals in each group for CSF analysis and only 4 AD and 3 normal in the immunohistochemistry experiments. Although the variable post-mortem time, would perhaps, not produce consistent results and may explain some of the variability we observed although published evidence suggests this should not be a significant issue [73]. Table 1 indicates the range in post-mortem times that were a consequence of the selection criteria for the study in terms of severity of AD. We wished to compare clearly neurologically normal brains to the most severe cases of AD within the Brain Bank to establish whether an effect existed or not. The immunohistochemical data is reported in a descriptive way due to the small number analysed.

## Figures and Tables

**Table 1:** (**a**) Normal ageing cases. (**b**) Alzheimer’s disease cases.

(a)								
Case No.	Braak Grade	MRC ID	Gender	Age at Death	Clinical Diagnosis	Pathological Diagnosis 1	Pathological Diagnosis 2	Post Mortem Delay (h)
DPM11/29	II	BBN_3467	M	89	normal	Age changes only	mild SVD	123
DPM13/07	0	BBN_10263	F	Na	normal	leptomeningeal Ca infiltration	Normal	90
DPM14/08	0-I	BBN_20005	M	85	Normal	Age changes only	moderate SVD	98
DPM14/11	I-II	BBN_20195	M	91	Normal	mild SVD	Na	43.5
DPM17/09	I	BBN005.30100	F	88	Control	Normal for age	ARTAG, possible PART	52.5
DPM17/36	I-II	BBN005.32382	F	94	Control	Age changes only	Na	70
DPM18/11	I-II	BBN005.32822	F	90	Control	Age changes only	Possible ARTAG	143
**DPM16/29**	**0-II**	**BBN005.29063**	**M**	**69**	**Control**	**Normal for age**		**53**
DPM16/30	0-II	BBN005.29167	F	95	Control	Normal for age/incipient AD	Moderate AD changes in temporal lobe	70.5
DPM16/31	0-II	BBN005.29168	M	90	Control	Normal for age	Mild SVD	155
DPM16/35	0-II	BBN005.29398	M	84	Control	Normal ageing	Mild to moderate SVD	41.5
DPM17/09	0-II	BBN005.30100	F	88	Control	Normal for age	ARTAG, possible PART	52.5
**DPM17/23**	**0-II**	**BBN005.30844**	**M**	**91**	**Control**	**Normal for age**	**Na**	**88**
**DPM17/36**	**0-II**	**BBN005.32382**	**F**	**94**	**Control**	**Age changes only**	**Na**	**70**
DPM17/38	0-II	BBN005.32526	F	101	Control	Normal for age	Na	135.5
DPM16/29	0-I	BBN005.29063	M	69	Control	Normal for age	Na	53
DPM18/03	0-I	BBN005.32560	M	88	Control	Normal for age	Na	39
DPM16/30	0-II	BBN005.29167	F	95	Control	Normal for age/incipient AD	Moderate AD changes in temporal lobe	70.5
DPM16/31	0-II	BBN005.29168	M	90	Control	Normal for age	Mild SVD	155
DPM16/35	0-II	BBN005.29398	M	84	Control	Normal ageing	Mild to moderate SVD	41.5
DPM17/23	0-II	BBN005.30844	M	91	Control	Normal for age	Na	88
DPM14/46	0-I	BBN_24316	F	94	control	age changes only	mild SVD	111
DPM16/30	II	BBN005.29167	F	95	Control	Normal for age/incipient AD	Na	70.5
DPM16/35	II	BBN005.29398	M	84	Control	Normal ageing	Na	41.5
DPM13/07	II	BBN_10263	F	60	normal	leptomeningeal Ca infiltration	Na	90
DPM14/34	0	BBN_24212	F	94	control	age changes only	Na	74.5
(**b**)								
**Case No.**	**Braak Grade**	**MRC ID**	**Gender**	**Age at Death**	**Clinical Diagnosis**	**Pathological Diagnosis 1**	**Pathological Diagnosis 2**	**Post Mortem Delay (h)**
DPM11/28	VI	BBN_3466	F	71	Alzheimer’s disease	Alzheimer’s disease		5
DPM12/01	V-VI	BBN_3469	M	67	Dementia	Alzheimer’s disease	mild SVD	84
DPM12/03	VI	BBN_3470	M	72	Alzheimer’s Disease	Alzheimer’s disease	Na	81
DPM12/05	VI	BBN_3472	M	73	Alzheimer’s Disease	Alzheimer’s disease	mod SVD	5
DPM12/25	V-VI	BBN_6076	M	62	Semantic Dementia	Alzheimer’s disease	Na	50.5
DPM12/32	V-VI	BBN_9480	M	73	Alzheimer’s disease	Alzheimer’s disease	Na	26
DPM13/10	V-VI	BBN_11028	F	85	dementia	Alzheimer’s disease	Mild CAA	24
DPM14/10	V-VI	BBN_20007	F	78	Alzheimer’s disease	Alzheimer’s disease	CAA with capillary involvement	70
DPM14/30	V-VI	BBN_23794	F	70	dementia, learning difficulty	Alzheimer’s disease	Na	89
DPM14/31	VI	BBN_23803	M	64	Alzheimer’s Disease	Alzheimer’s disease	moderate SVD	98.5
**DPM14/50**	**V-VI**	**BBN_24361**	**F**	**63**	**Alzheimer’s Disease**	**Alzheimer’s disease**	**moderate SVD**	**54**
**DPM15/02**	**V-VI**	**BBN_24373**	**M**	**78**	**Alzheimer’s Disease**	**Alzheimer’s disease**	**sec TDP-43 proteinopathy**	**173**
DPM15/29	V-VI	BBN_25921	M	81	AD and Vascular dementia	Alzheimer’s disease	mild SVD	68
**DPM16/10**	**V-VI**	**BBN005.28400**	**F**	**59**	**Alzheimer’s Disease**	**Alzheimer’s disease**	**Na**	**87**
**DPM18/27**	**V-VI**	**BBN005.33712**	**M**	**75**	**Alzheimer’s Disease**	**Alzheimer’s disease**	**Na**	**104.5**
DPM16/40	VI	BBN005.29461	M	82	Alzheimer’s Disease	Alzheimer’s disease	Moderate CAA	25.5
DPM17/37	V-VI	BBN005.32384	F	90	Alzheimer’s Disease	Alzheimer’s disease	Possible AGD	76
DPM18/12	VI	BBN005.32823	M	70	Alzheimer’s Disease	Alzheimer’s disease	Moderate SVD	120.5
DPM18/39	VI	BBN005.35131	F	75	Dementia	Alzheimer’s disease	Na	127.5
DPM19/04	VI	BBN005.35211	M	82	Alzheimer’s Disease	Alzheimer’s disease	Temporal intra-cortical infarct. Secondary TDP-43.	124
DPM19/07	VI	BBN005.35399	F	86	Dementia	Alzheimer’s disease	Severe hippocampal sclerosis. Secondary TDP-43.	72
DPM20/07	VI	BBN005.36133	F	88	Alzheimer’s Disease	Alzheimer’s disease	Na	165.5
DPM10/15	VI	BBN_5766	F	76	Alzheimer’s disease	Alzheimer’s disease	Na	52
DPM14/10	V-VI	BBN_20007	F	78	Alzheimer’s disease	Alzheimer’s disease	Na	70
DPM11/28	VI	BBN_3466	F	71	Alzheimer’s disease	Alzheimer’s disease	Na	64
DPM12/03	VI	BBN_3470	M	72	Alzheimer’s Disease	Alzheimer’s disease	Na	81
DPM12/17	VI	BBN_6068	M	76	Alzheimer’s Disease	Alzheimer’s disease	Na	96
DPM14/31	VI	BBN_23803	M	64	Alzheimer’s Disease	Alzheimer’s disease	Na	98.5
DPM12/01	V-VI	BBN_3469	M	67	Dementia	Alzheimer’s disease	Na	84
DPM14/21	IV	BBN_21006	M	72	FTD	Alzheimer’s disease	Na	103
DPM13/30	IV	BBN_15257	M	77	FTD/PNFA	Alzheimer’s disease	Na	87

Only individuals who were neuropathologically normal (Braak 0-II, *n =* 21), based on clinical observation and post-mortem neuropathology assessment, or with severe AD (Braak V-VI, *n =* 21) were included in this small cohort study. Due to these selection criteria, there was significant variability in post-mortem times to collection of tissue (Table 1). The CSF from all cases was analysed but for tissue analysis and immunohistochemistry we used 3 cases from neurologically normal and 4 from severe AD (highlighted in bold text in Table). A full analysis of clinic-pathological features for these patients has been published in our previous report [20]. In a study addressing the issues of post-mortem delay (PMD) to the quality of brain tissue samples, Robinson et al. [73] refute the view that extended PMD is detrimental to brain tissue quality. The paper indicates that this is determined by brain pH and that pH is not significantly affected by post-mortem time.

**Table 2:** Antibodies used in this study.

Primary Antibodies				Dilution		
Target	Species	Source	Ref. No	WB	DB	IHC
FDH	Rb anti Human	S.Krupenko	Gift	---	---	1:1000
FDH	Ms-anti-ALDH1L1	Sigma	SAB 4100141	---	---	1:1000
FRα	Gt-anti-FOLR1	R & D System	AF5646	1:3000	---	1:1000
Folates	Ms anti Human	Sigma	M-5028	---	---	---
GFAP	Chkn-anti-GFAP	Encor	CPCA-GFAP	---	---	1:3000
S100	Rb-anti-S100	Atlas Ab	HPA015768	---	---	1:1000
5-HmCyt	Rat-anti-5-HmCyt	Abcam	Ab106918	---	---	1:1000
5-mCyt	Rb-anti-5mCyt	Cell Signalling	D3S2Z	---	---	1:1000
**Fluorophore-Conjugated Secondary Antibodies for WB/DB**				**Dilutions**		
**Species**		**Source**	**Ref. No**	**WB**		**DB**
anti Rb		Bio-Rad	10000068188D	1:3000		----
anti Ms		Bio-Rad	10000086250B	1:3000		1:3000
**Alexa Fluor Secondary Antibodies for IHC**						**Dilutions**
**Species**		**Source**	**Ref. No**		**wavelength**	**IHC**
Anti-Chkn		Abcam	ab150176		594	1:3000
Anti-Rb		Abcam	ab150077		488	1:1000
Anti-Ms		Abcam	ab150120		594	1:1000
Anti-Gt		Abcam	ab150132		594	1:1000
Anti-Ms		Abcam	ab150113		488	1:1000
Anti-Chkn		Abcam	ab150169		488	1:3000
Anti-Rat		Abcam	ab150165		488	1:1000
Anti-Rb		Abcam	ab150080		594	1:1000
Anti-Rb		Abcam	ab150075		647	1:1000
Anti-Gt		Abcam	ab150129		488	1:1000
Anti-Ms		Abcam	ab150110		555	1:1000

## Data Availability

All data are available from the corresponding author.

## Supplementary Materials

The supporting information can be downloaded at: https://www.mdpi.com/article/10.3390/ijms24010660/s1.

## Abbreviations

AD	Alzheimer’s disease
FOLR1 and FRα	folate receptor alpha
ALDH1L1 and FDH	10-formyl tetrahydrofolate dehydrogenase
MTR	methionine synthase with full name: 5-methyltetrahydrofolate-homocysteine methyltransferase
MTRR	methionine synthase reductase, full name: methionine synthase-cobalamin methyltransferase
MTHFD1	methylene tetrahydrofolate dehydrogenase 1
DHFR	dihydrofolate reductase
MTHFR	methylene tetrahydrofolate reductase
5mTHF	5 methyl tetrahydrofolate
GFAP	glial fibrillary acidic protein
5mCyt	5 methyl cytosine
5HmCyt	5 hydroxy methyl cytosine
