# Supplementary material for: Cerebral Folate Metabolism in Post-Mortem Alzheimer’s Disease Tissues: A Small Cohort Study
Source: Int J Mol Sci. 2022 Dec 30;24(1):660. doi: 10.3390/ijms24010660 (PMC9820589; doi:10.3390/ijms24010660)
Supplement: Supplementary file 1 [file ijms-24-00660-s001.zip › ijms-2085580-supplementary.pdf]

Supplementary Figure S1a

Folate dot blots

Braak 0-II

Braak 0-II

Braak V-VI

Braak V-VI

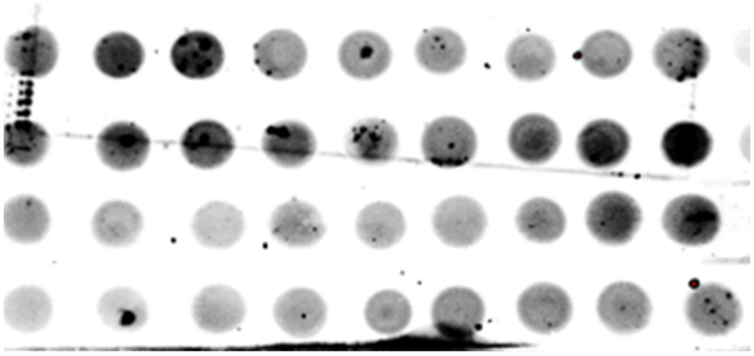

Lane 1 11/29 13/07 14/11 14/08 14/34 10/15 11/26 12/25 12/07 12/25

Lane 2 16/32 16/30 16/30 16/15 16/30 12/17 12/03 13/30 13/10 14/21

Lane 3 16/15 16/23 16/30 16/15 17/36 14/07 14/30 14/31 14/10 14/50

Lane 4 17/7 18/11 17/23 17/09 15/02 15/29 16/10 18/27 20/07

Dot blots to determine folate content of CSF: CSF was mixed 1:1 with Laemmle buffer and then heated to 70C. 6ul was pipetted onto the nitrocellulose membrane, allowed to dry and then probed as for western blots.

Supplementary Figure S1b

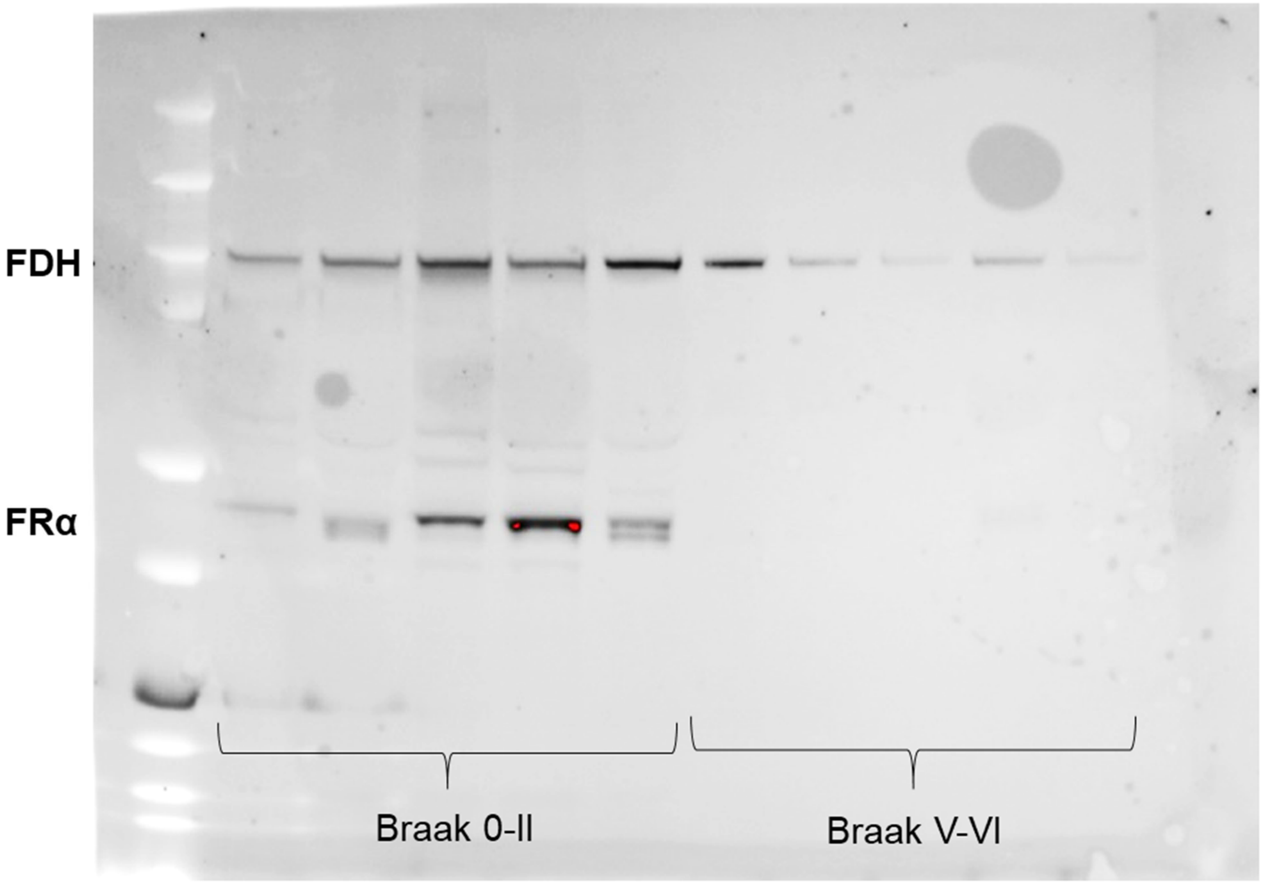

Western blots for FDH and FRα in CSF.

Both primary antibodies were applied to the same membrane as they are easily distinguished by molecular weight. This saves sample volume.

Supplementary Figure S2

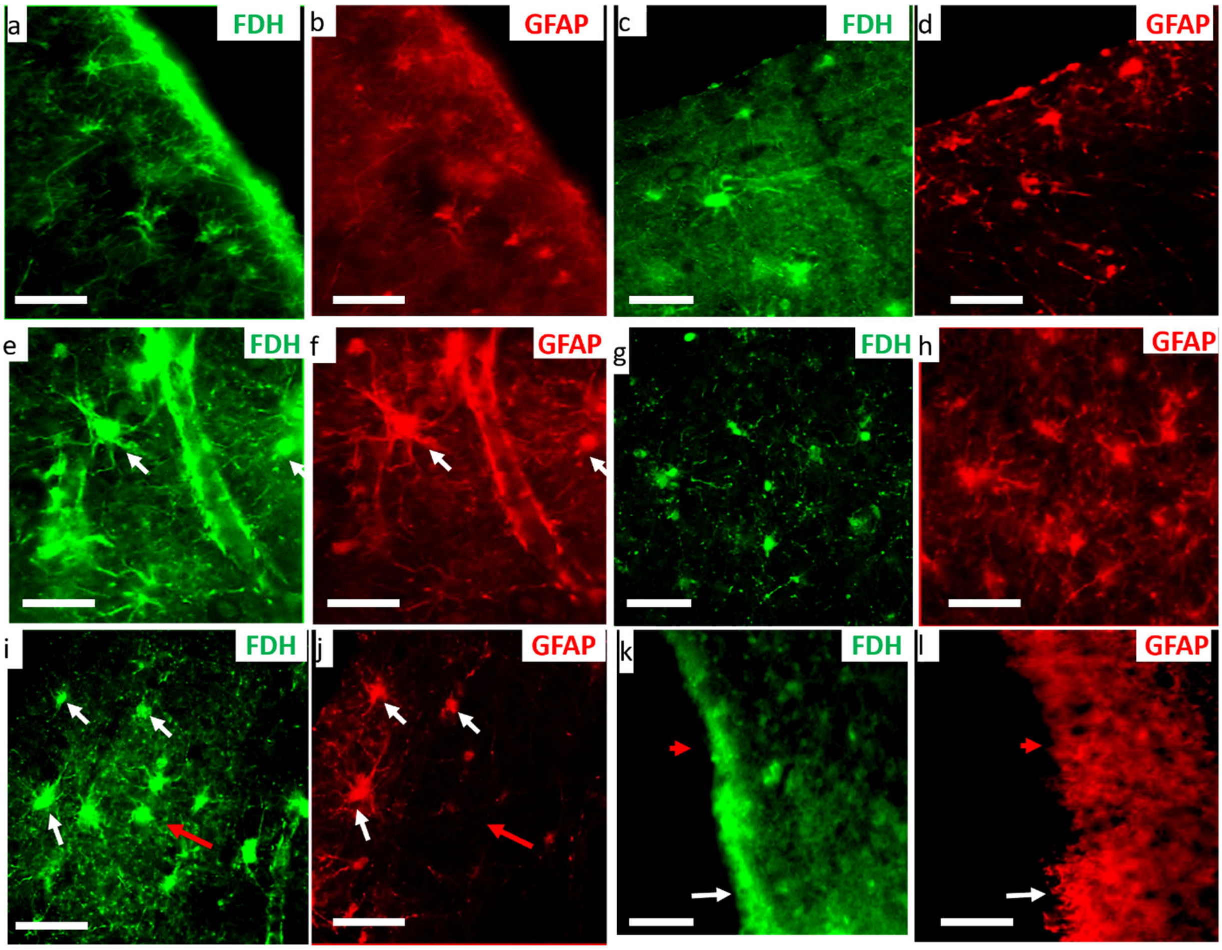

Immunofluorescence staining of Normal (Braak0-II) brain sections. Top row shows staining in cortical regions near the pia mater (100x magnification, scale bars 100 µm), Centre row shows white matter regions while bottom row shows sub ventricular zone (I,j) and ventricular zone (k,l) at 200x magnifications, scale bars 50 µm. The white arrows represent $FDH^+/GFAP^+$ whereas red arrows indicate $FDH^+/GFAP^-$. The figure is representative of neurologically normal brains, n=3.

Supplementary Figure S3

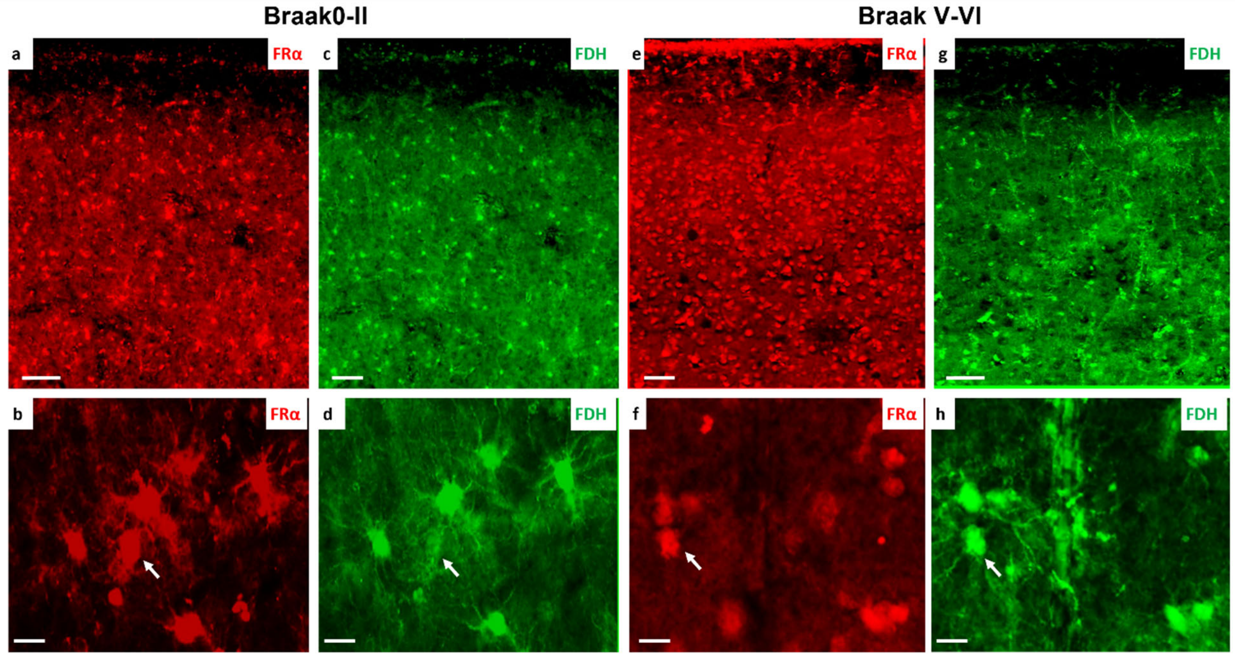

Immunofluorescence staining for FRα and FDH in normal (Braak 0-II) and AD (Braak V-VI) brain sections (a,c,e,g. 100x,100μm and b,d,f,h, 400x, 20 μm). FRα is colocalised with FDH in cells with astrocyte morphology with some exceptions, e.g. white arrow in b is not FDH positive in d. The cells labelled with the white arrow in f and h are dual stained but the FRα is limited to the soma while the processes are FRα negative. Compare this to the complete colocalization in normal brain. The figure is representative of neurologically normal n=3 and AD brains n=4.

Supplementary Figure S4

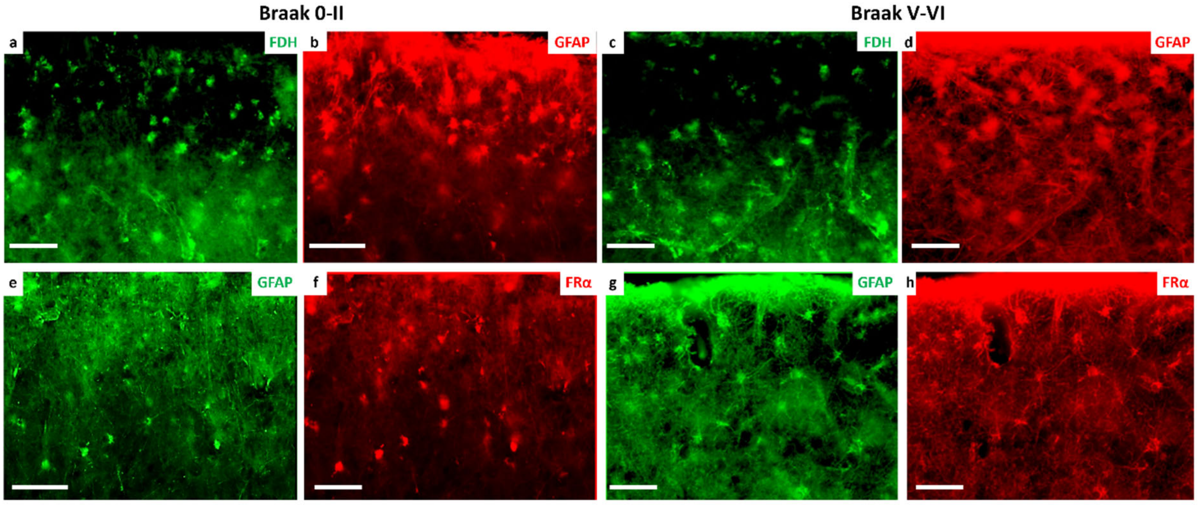

Immunofluorescence staining for FDH, GFAP and FRα. d-d FDH (green) and GFAP (red). e-h GFAP (green) and FRα (red). In both normal (a,b) and AD (c,d) brains FDH and GFAP are largely separated. GFAP and FRα are also largely separated in normal (e,f) but are clearly colocalised in AD (g,h) brain. This indicates a switch in folate transport with FRα moving from the FDH positive astrocyte pathway in normal to the GFAP positive pathway in AD. Magnification 200x, scale bar 50 μm. The figure is representative of neurologically normal n=3 and AD brains n=4.

Supplementary Figure S5

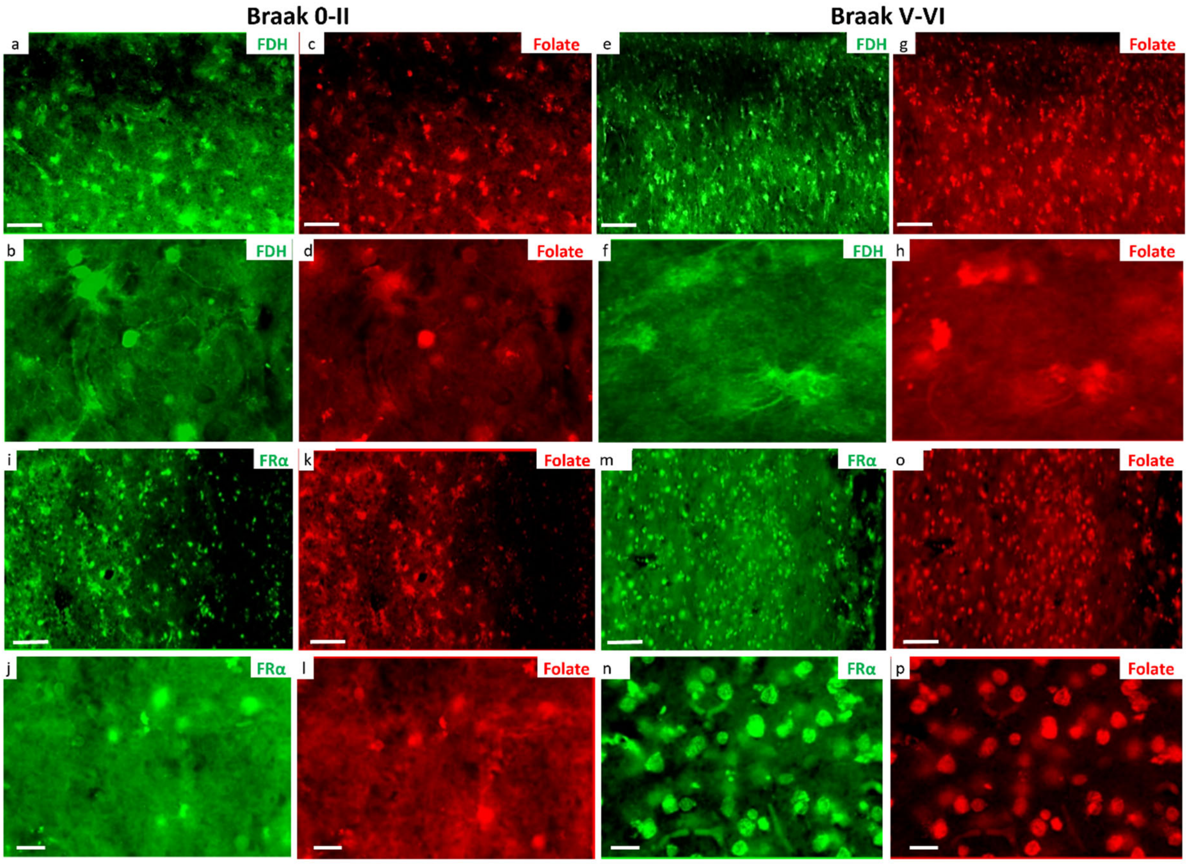

Immunofluorescence staining for FDH (a,b,e,f), FRα (i,j,m,n) and folate (c,d,g,h,k,l,o,p) in normal (Braak 0-II) and AD (Braak V-VI) brain sections (row 1 and 3are 100x 100µm, row 2 and 4 are 400x 20µm). There is generally good colocalization of folate in FDH positive cells (a,c) with variable amounts of folate seen in some cells (b,d) in normal brain, whichIn normal brain FRα is colocalised with folate in FDH positive cells but in AD brain FRα is found in nuclei of neurones along with folate as well as in GFAP positive cells (see main paper for these data). The figure is representative of neurologically normal n=3 and AD brains n=4.

Supplementary Figure S6

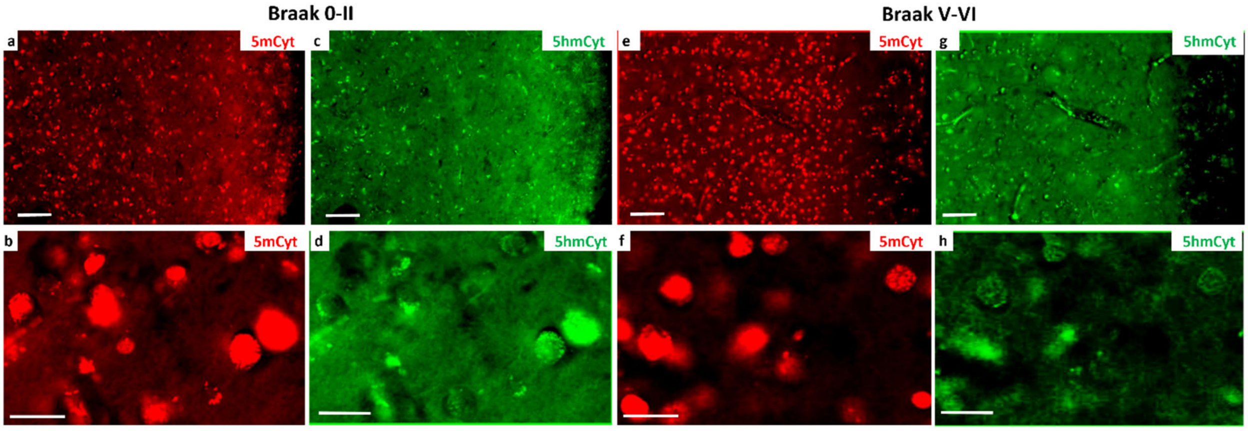

Immunofluorescence staining for 5-methyl cytosine (a,b,e,f) and 5-hydroxy methyl cytosine (c,d,g,h) in normal (Braak 0-II) and AD (Braak V-VI) brain sections. Top row is at 100x 100µm and bottom row is ay 400x 20µm. The figure is representative of neurologically normal n=3 and AD brains n=4.
